# Suppressive Effects of Cooling Compounds Icilin on Penicillin G-Induced Epileptiform Discharges in Anesthetized Rats

**DOI:** 10.3389/fphar.2019.00652

**Published:** 2019-06-13

**Authors:** Hiroshi Moriyama, Sadahiro Nomura, Hiroyuki Kida, Takao Inoue, Hirochika Imoto, Yuichi Maruta, Yuichi Fujiyama, Dai Mitsushima, Michiyasu Suzuki

**Affiliations:** ^1^Department of Neurosurgery, Graduate School of Medicine, Yamaguchi University, Yamaguchi, Japan; ^2^Epilepsy Center, Yamaguchi University Hospital, Yamaguchi, Japan; ^3^Department of Physiology, Graduate School of Medicine, Yamaguchi University, Yamaguchi, Japan

**Keywords:** focal epilepsy, epileptiform discharges, transient receptor potential melastatin 8 channel, icilin, cortical temperature, rat

## Abstract

More than 30% of patients with epilepsy are refractory and have inadequate seizure control. Focal cortical cooling (FCC) suppresses epileptiform discharges (EDs) in patients with refractory focal cortical epilepsy. However, little is known about the mechanism by which FCC inhibits seizures at 15°C, and FCC treatment is highly invasive. Therefore, new antiepileptic drugs are needed that produce the same effects as FCC but with different mechanisms of action. To address this need, we focused on transient receptor potential melastatin 8 (TRPM8), an ion channel that detects cold, which is activated at 15°C. We examined whether TRPM8 activation suppresses penicillin G (PG)-induced EDs in anesthetized rats. Icilin, a TRPM8 and TRP Ankyrin 1 agonist, was administered after PG injection, and a focal electrocorticogram (ECoG) and cortical temperature were recorded for 4 h. We measured spike amplitude, duration, firing rate, and power density in each band to evaluate the effects of icilin. PG-induced EDs and increased delta, theta, alpha, and beta power spectra were observed in the ECoG. Icilin suppressed EDs while maintaining cortical temperature. In particular, 3.0-mM icilin significantly suppressed PG-induced spike amplitude, duration, and firing rate and improved the increased power density of each band in the EDs to the level of basal activity in the ECoG. These suppressive effects of 3.0-mM icilin on EDs were antagonized by administering N-(3-aminopropyl)-2-[(3-methylphenyl) methoxy]-N-(2-thienylmethyl)-benzamide hydrochloride (AMTB), a selective TRPM8 inhibitor. Our results suggest that TRPM8 activation in epileptic brain regions may be a new therapeutic approach for patients with epilepsy.

## Introduction

The lifetime prevalence of epilepsy is 7.60 per 1,000 people (Fiest et al., 2017), and more than 30% of patients with epilepsy have inadequate control of seizures (Kwan and Brodie, 2000). Because the percentage of patients with epilepsy who develop refractory epilepsy positively correlates with the number of seizures before treatment with antiepileptic drugs (Kwan and Brodie, 2000), it is important to reduce the number of seizures to prevent refractory epilepsy. Given these issues, we proposed that refractory epilepsy can be treated by focal cortical cooling (FCC) to 15°C to 25°C (Nomura et al., 2014). However, little is known about the mechanism by which cooling inhibits seizures. In addition, treatment using FCC is highly invasive; therefore, new antiepileptic drugs involved in cooling but with different mechanisms of action are required to provide options to antiepileptic treatment using FCC.

Considering the antiepileptic effects of FCC, we focused on transient receptor potential (TRP) channels. The physiological function of TRP channels depends on their activation in a specific temperature range (Clapham, 2003). The TRP melastatin 8 (TRPM8) channel is activated by cold (10°C–26°C) (Bautista et al., 2007) or by TRPM8 agonists, such as icilin, a cooling compound with high affinity for TRPM8 (McKemy et al., 2002). Icilin, a TRPM8 and TRP Ankyrin 1 agonist, reduced the amplitude of glutamatergic excitatory postsynaptic currents (EPSCs) in an electrophysiological study (Wrigley et al., 2009). These reports suggest that TRPM8 activation is involved in the suppressive effect of FCC on epilepsy. However, the antiepileptic effects of TRPM8 activation in the somatosensory cortex are unknown.

Penicillin G (PG)-induced experimental epilepsy is a common model of epileptic activity mediated by γ-aminobutyric acid (GABA)_A_-receptor antagonists (Schwartzkroin and Prince, 1977; Fisher, 1989; Bertsche et al., 2010; Kida et al., 2012), and PG injection at a dose of 1,200 IU is a model of epileptic focal seizures (Fujii et al., 2012). Epileptiform discharges (EDs) have been detected both ictal and interictal electroencephalogram (Manoochehri et al., 2017), and focal cortical dysplasias are associated with focal epileptic discharges of variable morphologies in the beta frequency band in addition to single epileptic spikes (Heers et al., 2014). Therefore, we hypothesized that if TRPM8 activation suppresses the increased power density of each band including the beta band, TRPM8 agonists may be new antiepileptic drug options. In this study, using electrocorticogram (ECoG) recordings, we examined whether TRPM8 activation by icilin suppresses EDs which were induced by administrated PG in the somatosensory cortex during anesthetized rats.

## Methods

Male Sprague–Dawley rats aged 10 to 11 weeks and weighing 320 to 400 g (Japan SLC Inc. Fukuoka, Japan) were housed in groups of three rats per cage and kept under standard laboratory conditions in a temperature- and humidity-controlled room (23 ± 2°C, 55 ± 5%, respectively) on a 12-h light/dark cycle (lights on at 8:00 a.m). The animals had free access to food and water. The animal care and experimental procedures were approved by the Experimental Animal Care and Use Committee of Yamaguchi University School of Medicine, Japan. All experiments were performed in accordance with the guidelines of the Japan Association for Laboratory Animal Facilities of National University Corporations.

The animals were anesthetized with urethane (1.4 g/kg intraperitoneally) and immobilized using a stereotaxic apparatus. Each rat’s body temperature was measured rectally and adjusted to 37 ± 2°C using a heating pad. We maintained cortical temperature at 35 ± 2°C (**Supplementary Table S1**). We created two burr holes measuring 1.0-mm diameter, one for the ECoG recording and one for the reference electrode, above bilateral sensorimotor cortices and the cerebellum using the following coordinates relative to the bregma and the lambda: posterior, 2.0 mm; lateral, 4.0 mm; and posterior, 2.0 mm. We also placed a thin thermocouple (IT-24; Physitemp, Tokyo, Japan), and two ECoG and reference electrodes in bilateral somatosensory cortices and the cerebellar area between the skull and dura. We placed a ground electrode in the tail. A small slit in the dura was made to insert an injection cannula (0.4 mm diameter × 40 mm length, and 3 µl volume; EIM-40; Eicom, Kyoto, Japan). PG (Meiji, Tokyo, Japan) and icilin (Merck KGaA, Darmstadt, Germany) were injected into the same location *via* the same route without mixing, using the following procedure: first, we used a 10-µl Hamilton syringe with a 26-gauge removable needle (1701RN-7758-02; Hamilton, Reno, NV). The 1-µl inner cavity of the removable needle was filled with 1 µl of PG (dissolved in 0.9% saline at 400 IU/µl). The 10-µl syringe body was filled with 1 µl of PG and 8.8 µl of icilin, which were separated by 0.2 µl of air. PG and N-(3-aminopropyl)-2-[(3-methylphenyl) methoxy]-N-(2-thienylmethyl)-benzamide hydrochloride (AMTB) were also separated by 0.2 µl of air. Second, the injection cannula and Hamilton syringe were connected through a Teflon tube (JT-10; 50-cm-long, 4-µl volume; Eicom). After the PG filled in the Hamilton syringe reached the tip of the injection cannula through the Teflon tube, the injection cannula was inserted to a depth of 1 mm from the brain surface. PG was administered intracortically for 10 min at a rate of 0.1 µl/min using a microinjection pump (ESP-64, Eicom, Japan), beginning 60 min after the start of the ECoG recording.

Icilin [dissolved in 1% dimethyl sulfoxide (DMSO: Merck KGaA) in saline] was administered intracortically for 10 min, with icilin administration starting 90 min after the PG injection, at a rate of 0.1 µl/min using a microinjection pump. The spike amplitudes of PG-induced EDs with reference to baseline were averaged every 10 min. Because the duration in which the ED amplitude was statistically suppressed was considered the period of drug efficacy, we selected a duration from 100 to 110 min after PG injection as the postinjection period (**Supplementary Figure S2**). ECoG activities with preadministration of AMTB were averaged over 10 min, from 70 to 80 min after PG injection.

ECoGs were amplified by a bio-amplifier (EX-1; Dagan Corporation, Minneapolis, MN) and continuously recorded for 4 h (1 h for ECoG stabilization and 3 h for acquisition of PG-induced EDs) using an analogue/digital converter at a sampling rate of 2 kHz (PowerLab 8/30; AD Instruments, Castle Hill, Australia). The conditions for recording ECoGs were as follows: low-frequency filter, 0.1 Hz; high-frequency filter, 10 kHz; notch filter: off. We measured the following four parameters: spike amplitude (Tse et al., 2014), duration (Tse et al., 2014), power density of each band (Kida et al., 2012), and firing rate (Tse et al., 2014) using Lab Chart Pro v. 8.1.5 (AD Instruments). The spike amplitude and duration detected in each rat were automatically calculated and measured using this software, and after the ECoG was fast Fourier-transformed, the absolute band power was calculated for prominent ECoG spectral bands (delta, 1–4 Hz; theta, 4–9 Hz; alpha, 9–14 Hz; beta 1, 14–24 Hz; and beta 2, 24–30 Hz). To clarify whether icilin affects the ECoG in all frequency bands or in a specific frequency band, we calculated the power density of ECoG in each frequency bands during basal activity: the 10-min period just before PG injection, preinjection: control group for the efficacy evaluation, and postinjection: the 10-min period just after the end of the latest injection. Spike duration was defined as a spike wave with a duration < 100 ms (Kida et al., 2012). In this study, the rats were used independently in each group. Regarding the inhibition of TRPM8 by AMTB, we evaluated ECoG recordings using three different doses of AMTB for the treatment groups, namely, PG + 0.3 mM AMTB + 3.0 mM icilin, PG + 1.0 mM AMTB + 3.0 mM icilin, and PG + 3.0 mM AMTB + 3.0 mM icilin, and for the control groups, which received PG + 0.3 mM AMTB + 1% DMSO, PG + 1.0 mM AMTB + 1% DMSO, and PG + 3.0 mM AMTB + 1% DMSO.

All results are expressed as mean ± standard error of the mean (SEM). Differences between preinjection and postinjection in the same groups for spike amplitude, spike duration, and firing rate were assessed using paired *t*-tests. Differences in spike amplitude, spike duration, and firing rate between groups were assessed by Dunnett’s test, with *p* < 0.05 indicating significance. Differences between groups for the power density of each spectrum were assessed by one-way analysis of variance (ANOVA) followed by Tukey’s test, with *p* < 0.05 indicating significance.

## Results

### Suppressive Effects of Icilin on Epileptiform Discharges

**Figure 1:**
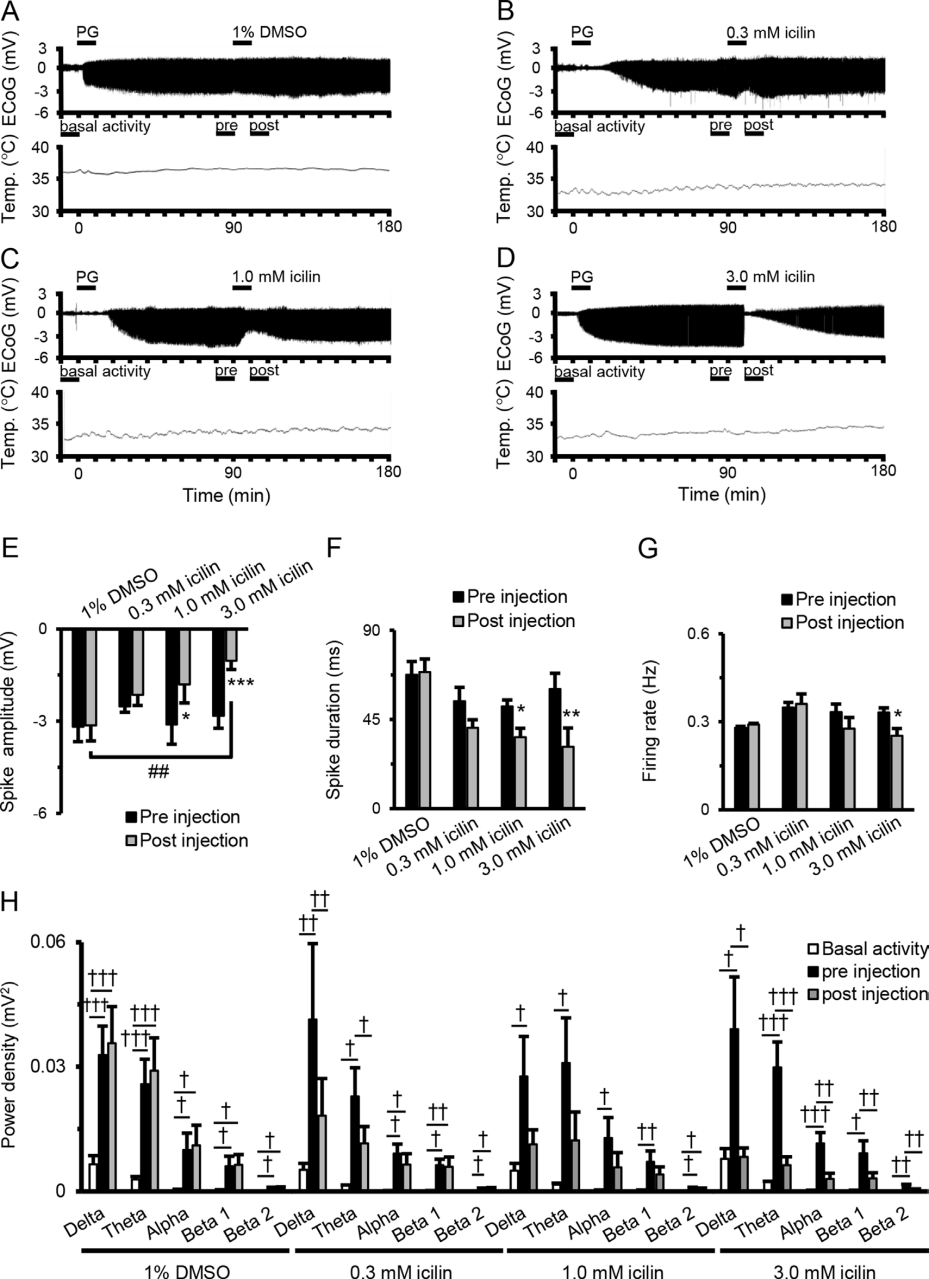
Suppressive effects of icilin on components of EDs. Examples of changes in ED and cortical temperature with **(A)** PG + 1% DMSO, **(B)** PG + 0.3 mM icilin, **(C)** PG + 1.0 mM icilin, **(D)** PG + 3.0 mM icilin. **(A–D)** Each bar indicates the duration of ECoG analysis during basal activity, and preinjection and postinjection. **(E)** Spike amplitude, **(F)** spike duration, and **(G)** firing rate during the preinjection (black) and postinjection (gray) periods with PG + 1% DMSO (*n* = 7), PG + 0.3 mM icilin (*n* = 7), PG + 1.0 mM icilin (*n* = 7), and PG + 3.0 mM icilin (*n* = 7). **(H)** ECoG power during basal activity (white), and the preinjection (black) and postinjection (gray) periods for each frequency band. The results are shown as mean ± SEM; *p < 0.05, **p < 0.01, ***p < 0.001, paired *t*-test; ^†^ *p* < 0.05, ^††^ *p* < 0.01, ^†††^ *p* < 0.001, one-way analysis of variance followed by Tukey’s test; ^##^ *p* < 0.01 vs PG + 1% DMSO group, followed by Dunnett’s test. ED, epileptiform discharge; PG, penicillin G; DMSO, dimethyl sulfoxide; ECoG, electrocorticogram; SEM, standard error of the mean.

**Figure 1A–D** shows examples of changes in ED, cortical temperature, and a typical ECoG preinjection and postinjection. The EDs were induced by the intracortically PG injection. There were no significant differences between EDs preinjection and postinjection of 1% DMSO (**Figure 1E and F**). There was a tendency for decreases in spike amplitude and duration after 0.3-mM icilin injection (**Figure 1E and F**), but the effect was not significant. There was a significant reduction in both spike amplitude and duration with 1.0 mM icilin (**Figure 1E and F**), but we saw no influence on firing rate (**Figure 1G**) or power density in each frequency band (**Figure 1H**). Compared with preinjection, 3.0 mM icilin eliminated all EDs for only a few minutes (**Figure 1D**) and significantly suppressed PG-induced spike amplitude, duration, firing rate, and power density in each frequency band in the EDs (**Figure 1E–H**). In particular, 3.0-mM icilin injection decreased the power density in each frequency band to the level of basal activity (**Figure 1H**).

### Suppressive Effects of Transient Receptor Potential Melastatin 8 Activation on Epileptiform Discharges

**Figure 2:**
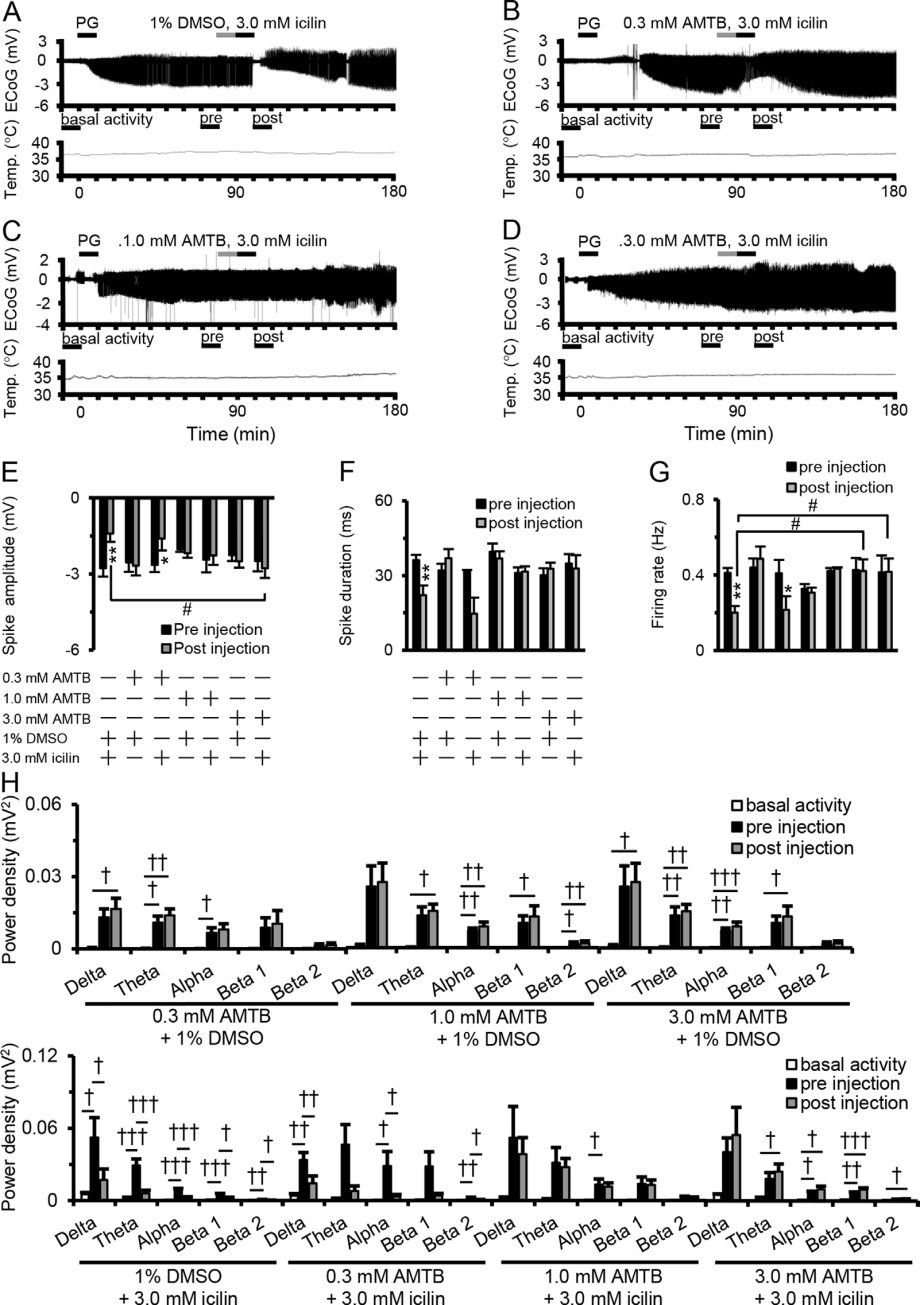
Antagonistic effect of AMTB on icilin-mediated ED suppression. Examples of changes in ED and cortical temperature with **(A)** PG + 1% DMSO + 3.0 mM icilin, **(B)** PG + 0.3 mM AMTB + 3.0 mM icilin, **(C)** PG + 1.0 mM AMTB + 3.0 mM icilin, and **(D)** PG + 3.0 mM AMTB + 3.0 mM icilin. **(A–D)** Each bar indicates the duration of ECoG analysis during basal activity, and preinjection and postinjection. **(E)** Spike amplitude, **(F)** spike duration, and **(G)** firing rate during the preinjection (black) and postinjection (gray) periods with PG + 1% DMSO + 3.0 mM icilin (*n* = 6), PG + 0.3 mM AMTB + 1% DMSO (*n* = 5), PG + 0.3 mM AMTB + 3.0 mM icilin (*n* = 5), PG + 1.0 mM AMTB + 1% DMSO (*n* = 5), PG + 1.0 mM AMTB + 3.0 mM icilin (*n* = 5), PG + 3.0 mM AMTB + 1% DMSO (*n* = 5), PG + 3.0 mM AMTB + 3.0 mM icilin (*n* = 6). **(H)** ECoG power during basal activity (white), and preinjection (black) and postinjection (gray) periods for each frequency band. The results are shown as mean ± SEM; **p < 0.01, paired *t*-test; ^†^ *p* < 0.05, ^††^ *p* < 0.01, ^†††^ *p* < 0.001, one-way analysis of variance followed by Tukey’s test; ^#^ *p* < 0.05 vs PG + 1% DMSO + 3.0 mM icilin group, followed by Dunnett’s test. AMTB, N-(3-aminopropyl)-2-[(3-methylphenyl) methoxy]-N-(2-thienylmethyl)-benzamide hydrochloride; ED, epileptiform discharge; PG, penicillin G; DMSO, dimethyl sulfoxide; SEM, standard error of the mean.

**Figure 2A–D** shows examples of changes in ED, cortical temperature, and a typical ECoG preinjection and postinjection. The suppressive effect of 3.0 mM icilin on PG-induced EDs such as increased spike amplitude, duration, firing rate, and power density in each frequency band (**Figure 1E–H**) was also detected when administering 3.0 mM icilin just after 1% DMSO injection (**Figure 2E–H**). However, the suppressive effect of 3.0 mM icilin on EDs was antagonized when N-(3-aminopropyl)-2-[(3-methylphenyl) methoxy]-N-(2-thienylmethyl)-benzamide hydrochloride (AMTB), a selective TRPM8 inhibitor, was administered at a dose of 3.0 mM just before icilin injection (**Figure 2E–H**). These results suggested that TRPM8 activation suppressed EDs.

## Discussion

To our knowledge, ours is the first report to show the suppressive effects of TRPM8 activation on EDs using ECoG analysis in a PG-induced epilepsy model in rats. Icilin decreased the spike amplitude, spike duration, firing rate, and all frequency bands in PG-induced EDs. These suppressive effects of 3.0 mM icilin were antagonized when AMTB was administered just before icilin injection. In this study, we did not use decreased brain surface temperature to activate TRPM8, which allowed us to reveal that TRPM8 activation by icilin suppressed the power density of each ED spectrum to the basal activity level (**Figure 1H**). FCC of the somatosensory motor area from 20°C to 15°C did not suppress normal function (Fujii et al., 2012), supporting our finding of the suppressive effects of icilin on PG-induced EDs without excessive suppression of basal ECoG activity.

The increase in power induced by PG in all bands was responsible for disinhibition of synchronous firing activity in the cortex, consistent with previous studies (Canan et al., 2008; Kida et al., 2012). An *in vivo* study showed that all bands (delta, theta, alpha, beta 1, and beta 2) in the power density spectra of PG-induced EDs were suppressed by FCC to 15°C (Kida et al., 2012), a temperature at which the TRPM8 channel is activated (Peier et al., 2002). In addition, a clinical report showed that FCC to 15°C also decreased the amplitude of EDs detected in an intraoperative study of patients with refractory epilepsy (Nomura et al., 2014). The firing rate of action potentials was also significantly lower after perfusion of 3.0 mM icilin (**Figure 1G**) and cooling to 15°C (Nomura et al., 2018). Administrated intracortically icilin at the dose of 3 µM suppressed the amplitude of EDs in C57BL/6 mouse, while administrated intracortically icilin at the dose of 3 µM did not affect the ECoG for PG-induced EDs in TRPM8 knockout (TRPM8KO) mouse (data not shown). In contrast with our conclusion, the previous report was concluded that icilin can modulate glutamatergic neurons in the brain through a TRP-independent pathway, regardless of whether the neurons are stimulated intracellularly or by hyperactive microcircuitry (Pezzoli et al., 2014). Moreover, it was reported that AMTB inhibited voltage gated sodium channels (Yapa et al., 2018). However, we revealed that the suppressive effect of high concentrated icilin on EDs was antagonized when AMTB, a TRPM8 inhibitor, was administered just before icilin injection (**Figure 2E–H**). We additionally examined that the effect of AMTB on the ECoG for basal activity and PG-induced EDs using C57BL/6 mice and TRPM8KO mice. Administrated intracortically AMTB at the dose of 3 µM did not affect the ECoG for basal activity and PG-induced EDs in C57BL/6 mice and TRPM8KO mice (Data not shown). These reports indicate that the suppressive effects of TRPM8 activation by icilin on focal cortical EDs are largely consistent with the reported physiological function of FCC. Moreover, the report by Pezzoli and our findings may suggest that the suppressive effects of icilin on EDs are not only TRPM8 activation but also through a TRP-independent pathway.

Our finding that 3.0 mM icilin reduced the duration of EDs does not agree with a report that showed that 50 µM icilin had no effect on action potential duration using normal cortical isolated neurons (Pezzoli et al., 2014). In contrast, our results describe the suppressive effects of high concentrated icilin on PG-induced EDs in the cortical region of rats when the neural network is maintained and cortical cells are abnormally synchronized by PG. These differences in measurement conditions could lead to the different results between the published data and our findings.

Icilin at the dose of 3.0 mM eliminated all EDs only for a few minutes (**Figure 1D**), whereas no EDs were detected when 3.0 mM icilin was administrated intracortically 30 min before PG injection (**Supplementary Figure S4**), and the previous study shown continuously eliminates EDs during the treatment of FCC at 15°C (Kida et al., 2012). The duration of elimination of EDs likely differs between TRPM8 activation and FCC mainly because of a difference in the number of suppression mechanisms for EDs. Icilin suppressed the amplitude of neuronal firing generated at a given voltage in layer V pyramidal neurons (Pezzoli et al., 2014), whereas menthol, a TRPM8 agonist, had a distinct impact on TRPM8 currents and TRPM8-mediated calcium signals in excitable cells (Janssens et al., 2016). Icilin reduced the amplitude of primary afferent-evoked glutamatergic excitatory postsynaptic currents (EPSCs) and increased miniature EPSC rates in subpopulations of lamina I and II neurons (Wrigley et al., 2009), whereas menthol produced a concentration-dependent extension of spontaneous GABA_A_ receptor-mediated inhibitory postsynaptic currents in native periaqueductal gray neurons (Lau et al., 2014). These reports indicate that TRPM8 activation temporarily but strongly suppresses EDs.

In addition, several reports have shown effects of FCC on EDs at a temperature range that induces TRPM8 activation. The amplitude of EDs induced by kainic acid, kainate type glutamate receptor agonist, was immediately decreased after cooling and continued to decrease as the temperature of the cortex was lowered (Imoto et al., 2006). Compared with 33°C, cooling to 20°C markedly reduced neurotransmitter release in rat hippocampal slices (Yang et al., 2005), and FCC to 15°C decreased the extracellular concentrations of glutamate (Nomura et al., 2014). In contrast, the reduction in the extracellular GABA by the treatment of FCC at 15°C did not correlate with the reduction of power density in ECoG (Nomura et al., 2017). These reports suggested that the reduction in the extracellular glutamate may have contributed to further suppression of EDs. Therefore, a more understanding of the antiepileptic action by icilin requires further study whether icilin modulate the concentration of extracellular glutamate.

Our findings may suggest that TRPM8 activation is involved in the suppressive effect of FCC on EDs. However, because urethane dramatically changes the background electroencephalogram, a more understanding of the antiepileptic effect by icilin requires further studies, the effects of icilin on seizures in awake subjects using TRPM8KO mice and rats are recorded ECoG by anesthetized with inhalation. In addition, a more complete understanding of this antiepileptic effect requires further studies: the expression of TRPM8 channels in the somatosensory cortex, the effects of TRPM8 inactivation under cooling conditions on EDs, the effects of TRPM8 activation on sensorimotor functions, and the effects of TRPM8 activation in chronic phase on EDs using TRPM8KO mice. In conclusion, the role of TRPM8 activation by icilin in suppressing EDs may be the basis for developing new drug treatment for patients with epilepsy.

## Ethics Statement

The animal care and experimental procedures were approved by the Experimental Animal Care and Use Committee of Yamaguchi University School of Medicine, Japan. All experiments were performed in accordance with the guidelines of the Japan Association for Laboratory Animal Facilities of National University Corporations.

## Author Contributions

HM conceived and designed the study, interpreted the data, and wrote the manuscript. HI, YM, and YF contributed to the conception of the study and interpreted the data. MS supervised the entire project. SN, HK, TI, and DM discussed the results and implications and commented on the initial manuscript. HM and SN revised the manuscript and the final version, which all authors reviewed and approved for publication.

## Funding

This work was supported by JSPS Kakenhi grants 15H05719 and 16H05438.

## Conflict of Interest Statement

The authors declare that the research was conducted in the absence of any commercial or financial relationships that could be construed as a potential conflict of interest.

## References

[B1] Bautista D. M. Siemens J. Glazer J. M. Tsuruda P. R. Basbaum A. I. Stucky C. L. (2007). The menthol receptor TRPM8 is the principal detector of environmental cold. Nature 448, 204–208. 10.1038/nature05910 17538622

[B2] Bertsche A. Bruehl C. Pietz J. Draguhn A. (2010). Region- and pattern-specific effects of glutamate uptake blockers on epileptiform activity in rat brain slices. Epilepsy Res. 88, 118–126. 10.1016/j.eplepsyres.2009.10.006 19939631

[B3] Canan S. Ankarali S. Marangoz C. (2008). Detailed spectral profile analysis of penicillin-induced epileptiform activity in anesthetized rats. Epilepsy Res. 82, 7–14. 10.1016/j.eplepsyres.2008.06.005 18657397

[B4] Clapham D. E. (2003). TRP channels as cellular sensors. Nature 426, 517–524. 10.1038/nature02196 14654832

[B5] Fiest K. M. Sauro K. M. Wiebe S. Patten S. B. Kwon C. S. Dykeman J. (2017). Prevalence and incidence of epilepsy: a systematic review and meta-analysis of international studies. Neurology 88, 296–303. 10.1212/WNL.0000000000003509 27986877 PMC5272794

[B6] Fisher R. S. (1989). Animal models of the epilepsies. Brain Res. Brain Res. Rev. 14, 245–278. 10.1016/0165-0173(89)90003-9 2679941

[B7] Fujii M. Inoue T. Nomura S. Maruta Y. He Y. Koizumi H. (2012). Cooling of the epileptic focus suppresses seizures with minimal influence on neurologic functions. Epilepsia 53, 485–493. 10.1111/j.1528-1167.2011.03388.x 22292464

[B8] Heers M. Hirschmann J. Jacobs J. Dümpelmann M. Butz M. von Lehe M. (2014). Frequency domain beamforming of magnetoencephalographic beta band activity in epilepsy patients with focal cortical dysplasia. Epilepsy Res. 108 (7), 1195–1203. 10.1016/j.eplepsyres.2014.05.003 24907181

[B9] Imoto H. Fujii M. Uchiyama J. Fujisawa H. Nakano K. Kunitsugu I. (2006). Use of a Peltier chip with a newly devised local brain-cooling system for neocortical seizures in the rat. J. Neurosurg. 104, 150–156. 10.3171/jns.2006.104.1.150 16509160

[B10] Janssens A. Gees M. Toth B. I. Ghosh D. Mulier M. Vennekens R. (2016). Definition of two agonist types at the mammalian cold-activated channel TRPM8. Elife 5, e17240. 10.7554/eLife.17240 27449282 PMC4985286

[B11] Kida H. Fujii M. Inoue T. He Y. Maruta Y. Nomura S. (2012). Focal brain cooling terminates the faster frequency components of epileptic discharges induced by penicillin G in anesthetized rats. Clin. Neurophysiol. 123, 1708–1713. 10.1016/j.clinph.2012.02.074 22459055

[B12] Kwan P. Brodie Mj. (2000). Early identification of refractory epilepsy. N. Engl. J. Med. 342, 314–319. 10.1056/NEJM200002033420503 10660394

[B13] Lau B. K. Karim S. Goodchild A. K. Vaughan C. W. Drew G. M. (2014). Menthol enhances phasic and tonic GABAA receptor-mediated currents in midbrain periaqueductal grey neurons. Br. J. Pharmacol. 171, 2803–2813. 10.1111/bph.12602 24460753 PMC4243856

[B14] Manoochehri M. Mahmoudzadeh M. Osharina V. Wallois F. (2017). Shedding light on interictal epileptic spikes: an *in vivo* study using fast optical signal and electrocorticography. Epilepsia 58 (4), 608–616. 10.1111/epi.13689 28117493

[B15] McKemy D. D. Neuhausser W. M. Julius D. (2002). Identification of a cold receptor reveals a general role for TRP channels in thermosensation. Nature 416, 52–56. 10.1038/nature719 11882888

[B16] Nomura S. Fujii M. Inoue T. He Y. Maruta Y. Koizumi H. (2014). Changes in glutamate concentration, glucose metabolism, and cerebral blood flow during focal brain cooling of the epileptogenic cortex in humans. Epilepsia 55, 770–776. 10.1111/epi.12600 24779587

[B17] Nomura S. Inoue T. Imoto H. Suehiro E. Maruta Y. Hirayama Y. (2017). Effects of focal brain cooling on extracellular concentrations of neurotransmitters in patients with epilepsy. Epilepsia 58, 627–634. 10.1111/epi.13704 28225164

[B18] Nomura S. Kida H. Hirayama Y. Imoto H. Inoue T. Moriyama H. (2018). Reduction of spike generation frequency by cooling in brain slices from rats and from patients with epilepsy. J. Cereb. Blood Flow Metab. 0 (00), 1–9. 10.1177/0271678X18795365. [Epub ahead of print]. PMC6827110 30117752

[B19] Peier A. M. Moqrich A. Hergarden A. C. Reeve A. J. Andersson D. A. Story G. M. (2002). A TRP channel that senses cold stimuli and menthol. Cell 108, 705–715. 10.1016/S0092-8674(02)00652-9 11893340

[B20] Pezzoli M. Elhamdani A. Camacho S. Meystre J. Gonzalez S. M. Le Coutre J. (2014). Dampened neural activity and abolition of epileptic-like activity in cortical slices by active ingredients of spices. Sci. Rep. 4, 6825. 10.1038/srep06825 25359561 PMC4215320

[B21] Schwartzkroin P. A. Prince D. A. (1977). Penicillin-induced epileptiform activity in the hippocampal *in vitro* preparation. Ann. Neurol. 1, 463–469. 10.1002/ana.410010510 617260

[B22] Tse K. Puttachary S. Beamer E. Sills G. J. Thippeswamy T. (2014). Advantages of repeated low dose against single high dose of kainate in C57BL/6J mouse model of status epilepticus: behavioral and electroencephalographic studies. PLoS One 9, e96622. 10.1371/journal.pone.0096622 24802808 PMC4011859

[B23] Wrigley P. J. Jeong H. J. Vaughan C. W. (2009). Primary afferents with TRPM8 and TRPA1 profiles target distinct subpopulations of rat superficial dorsal horn neurones. Br. J. Pharmacol. 157, 371–380. 10.1111/j.1476-5381.2009.00167.x 19371346 PMC2707984

[B24] Yang X. F. Ouyang Y. Kennedy B. R. Rothman S. M. (2005). Cooling blocks rat hippocampal neurotransmission by a presynaptic mechanism: observations using 2-photon microscopy. J. Physiol. 567, 215–224. 10.1113/jphysiol.2005.088948 15961429 PMC1474157

[B25] Yapa K. T. D. S. Deuis J. Peters A. A. Kenny P. A. Roberts-Thomson S. J. Vetter I. (2018). Assessment of the TRPM8 inhibitor AMTB in breast cancer cells and its identification as an inhibitor of voltage gated sodium channels. Life Sci. 198, 128–135. 10.1016/j.lfs.2018.02.030 29496495

