# Supplementary material for: Suppressive Effects of Cooling Compounds Icilin on Penicillin G-Induced Epileptiform Discharges in Anesthetized Rats
Source: Front Pharmacol. 2019 Jun 13;10:652. doi: 10.3389/fphar.2019.00652 (PMC6585232; doi:10.3389/fphar.2019.00652)
Supplement: Supplementary file 1 [file Table_1.docx]

Supplementary Table S1.

| **Table 1. Brain temperature during basal activity** **and pre- and postinjection** | | | |
| --- | --- | --- | --- |
| Group | Basal activity (°C) | Pre injection (°C) | Post injection (°C) |
| PG + 1% DMSO | 35.62 ± 0.53 | 35.88 ± 0.50 | 35.94 ± 0.50 |
| PG + 0.3 mM icilin | 34.01 ± 0.64 | 35.03 ± 0.33 | 34.88 ± 0.26 |
| PG + 1.0 mM icilin | 34.99 ± 0.66 | 35.16 ± 0.45 | 35.10 ± 0.43 |
| PG + 3.0 mM icilin  PG + 1% DMSO + 3.0 mM icilin  PG + 0.3 mM AMTB + 1% DMSO  PG + 0.3 mM AMTB + 3.0 mM icilin  PG + 1.0 mM AMTB + 1% DMSO  PG + 1.0 mM AMTB + 3.0 mM icilin  PG + 3.0 mM AMTB + 1% DMSO | 34.74 ± 0.60  35.52 ± 0.20  33.77 ± 0.20  34.44 ± 0.25  34.24 ± 0.29  33.67 ± 0.34  34.03 ± 0.18 | 35.37 ± 0.53  36.29 ± 0.13  35.06 ± 0.39  35.13 ± 0.11  35.44 ± 0.20  34.26 ± 0.27  35.25 ± 0.44 | 35.22 ± 0.54  35.99 ± 0.17  34.92 ± 0.36  35.49 ± 0.08  35.62 ± 0.36  34.48 ± 0.29  35.36 ± 0.37 |
| PG + 3.0 mM AMTB + 3.0 mM icilin | 35.30 ± 0.39 | 35.67 ± 0.31 | 35.74 ± 0.35 |
| PG, penicillin G; DMSO, dimethyl sulfoxide; AMTB, N-(3-aminopropyl)-2-[(3-methylphenyl)methoxy]-N-(2-thienylmethyl)-benzamide hydrochloride.  PG + 1% DMSO (n=7), PG + 0.3 mM icilin (n=7), PG + 1.0 mM icilin (n=7), PG + 3.0 mM icilin (n=7), PG + 1% DMSO + 3.0 mM icilin (n=6), PG + 0.3 mM AMTB + 1% DMSO (n=5), PG + 0.3 mM AMTB + 3.0 mM icilin (n=5), PG + 1.0 mM AMTB + 1% DMSO (n=5), PG + 1.0 mM AMTB + 3.0 mM icilin (n=5), PG + 3.0 mM AMTB + 1% DMSO (n=5), PG + 3.0 mM AMTB + 3.0 mM icilin (n=6). | | | |
