# Supplementary material for: Suppressive Effects of Cooling Compounds Icilin on Penicillin G-Induced Epileptiform Discharges in Anesthetized Rats
Source: Front Pharmacol. 2019 Jun 13;10:652. doi: 10.3389/fphar.2019.00652 (PMC6585232; doi:10.3389/fphar.2019.00652)
Supplement: Supplementary file 2 [file Image_1.pdf]

Supplementary Figure S1.

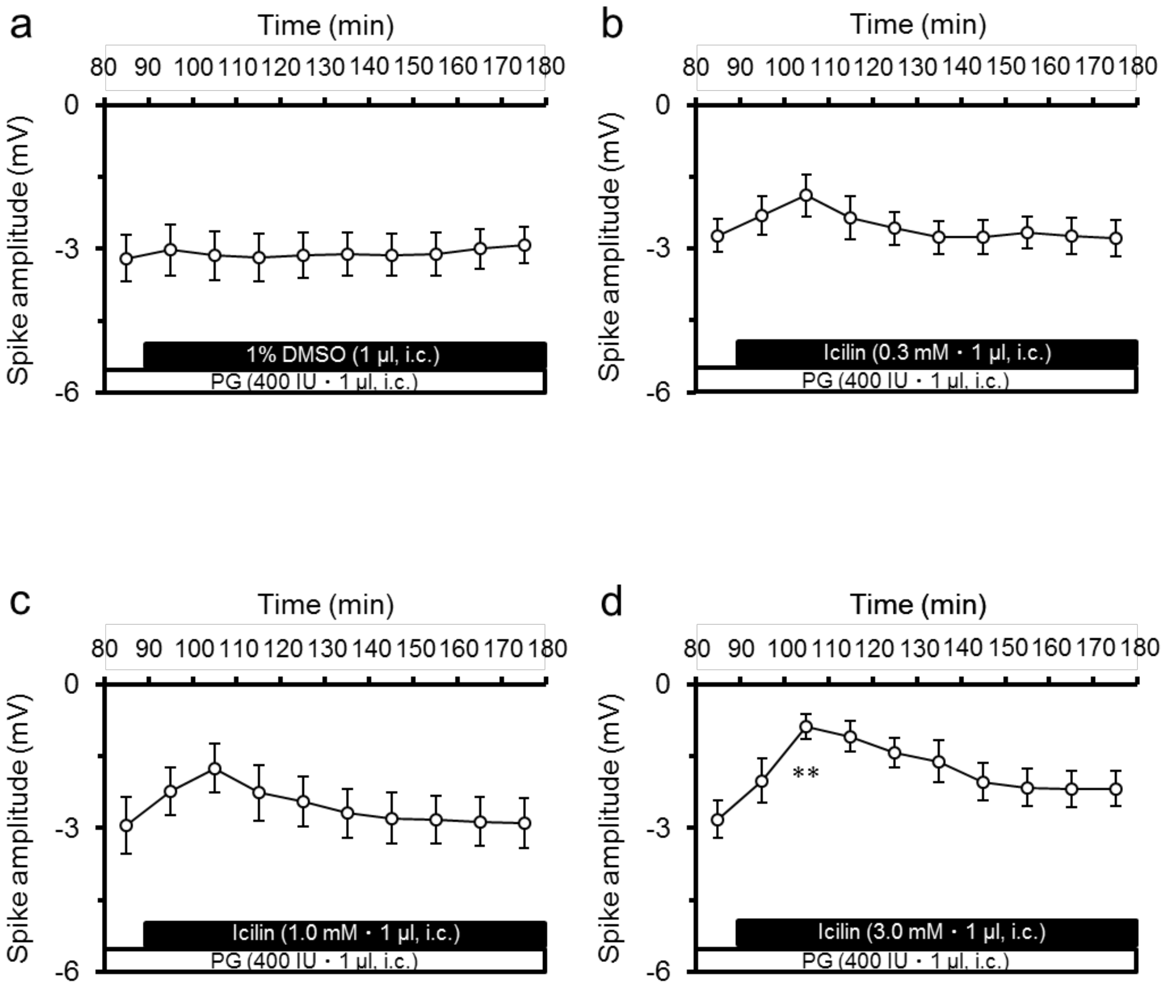

Supplemental Figure S1. Time courses of average spike amplitudes every 10 min from 80 min after PG injection. (a) PG + 1% DMSO (n = 7), (b) PG + 0.3 mM icilin (n = 7), (c) PG + 1.0 mM icilin (n = 7) and (d) PG + 3.0 mM icilin (n = 7). The results are shown as mean ± SEM; **p < 0.001 vs. 10 min average spike amplitude for the pre-injection period from 80 to 90 min using the Bonferroni test.
