# Supplementary material for: Suppressive Effects of Cooling Compounds Icilin on Penicillin G-Induced Epileptiform Discharges in Anesthetized Rats
Source: Front Pharmacol. 2019 Jun 13;10:652. doi: 10.3389/fphar.2019.00652 (PMC6585232; doi:10.3389/fphar.2019.00652)
Supplement: Supplementary file 3 [file Image_2.pdf]

Supplementary Figure S2.

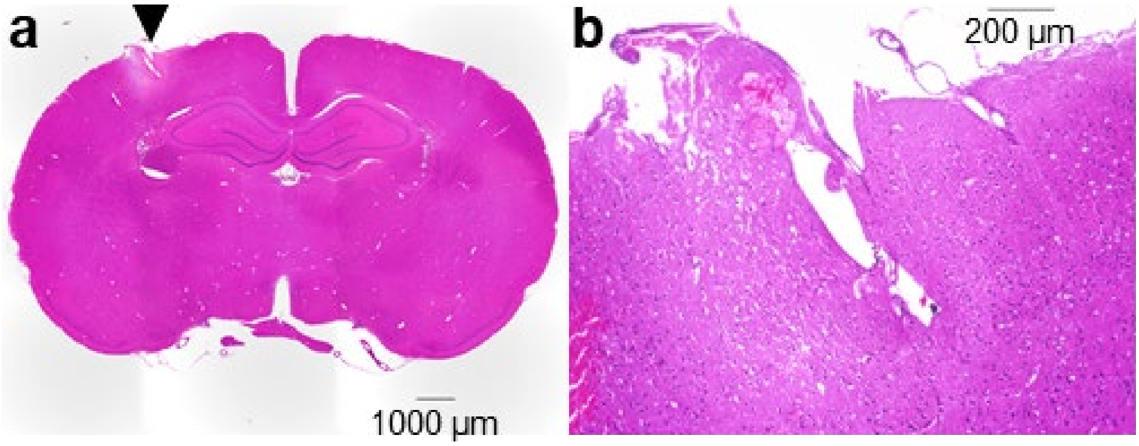

Supplementary Figure S2. (a) Photomicrographs of a coronal section from a rate in the PG + 3.0 mM icilin group acquired after the ECoG recording. The arrowhead shows the cannula injection location. Hematoxylin and eosin staining, scale bar = 1000 µm.

(b) Enlarged photomicrograph around the cannula injection location. Hematoxylin and eosin staining, scale bar = 100 µm.
