# Supplementary material for: Suppressive Effects of Cooling Compounds Icilin on Penicillin G-Induced Epileptiform Discharges in Anesthetized Rats
Source: Front Pharmacol. 2019 Jun 13;10:652. doi: 10.3389/fphar.2019.00652 (PMC6585232; doi:10.3389/fphar.2019.00652)
Supplement: Supplementary file 4 [file Image_3.pdf]

Supplementary Figure S3.

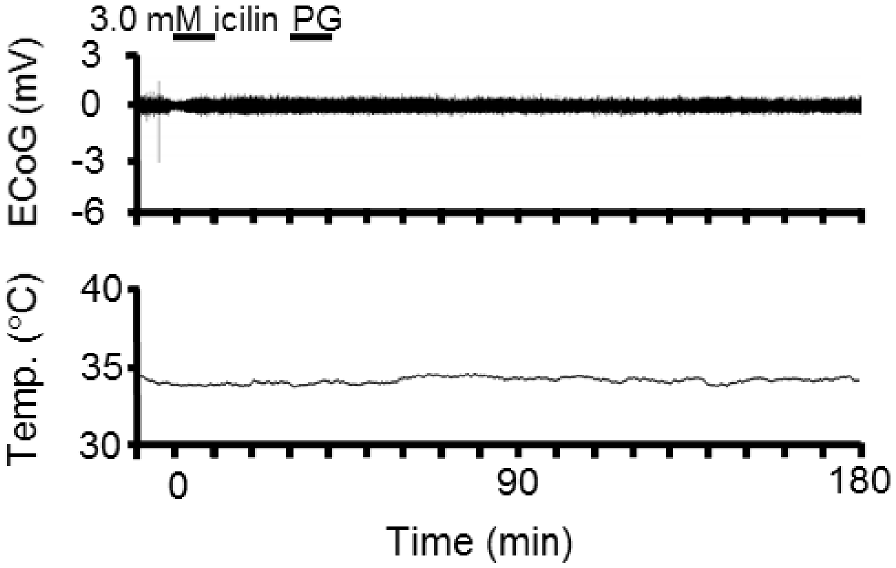

Supplemental Figure S3. Example of changes in ECoG and cortical temperature when 3.0 mM icilin was administrated intracortically 30 min before PG injection. Each bar indicates the duration for intracortically administrated 3.0 mM icilin and PG.
