# Supplementary figures and images for: Suppressive Effects of Cooling Compounds Icilin on Penicillin G-Induced Epileptiform Discharges in Anesthetized Rats
Source: Front Pharmacol. 2019 Jun 13;10:652. doi: 10.3389/fphar.2019.00652 (PMC6585232; doi:10.3389/fphar.2019.00652)

## Supplementary Figure S4

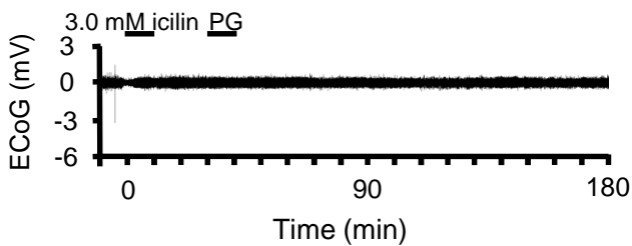

Supplement: Supplementary file 5 [file Image_4.pdf]
